# Phenotypic Variants of Azole-Resistant *Aspergillus Fumigatus* that Co-exist in Human Respiratory Samples are Genetically Highly Related

**DOI:** 10.1007/s11046-022-00665-2

**Published:** 2022-09-13

**Authors:** Alireza Abdolrasouli, Johanna L. Rhodes

**Affiliations:** 1 Department of Medical Microbiology, King’s College Hospital, London, UK; 2 MRC Centre for Global Disease Analysis, Imperial College London, London, UK

**Keywords:** *Aspergillus fumigatus*, Fungal culture, Phenotype, Whole-genome sequencing

## Abstract

Respiratory specimens obtained from patients with chronic forms of aspergillosis contain phenotypic variants of azole-resistant *Aspergillus fumigatus* (ARAF) that co-exist in the airway. Here we aimed to study whether phenotypic variants of ARAF that co-exist in clinical specimens were genetically distinct. A panel of six phenotypic variants of ARAF cultured from two sputum samples collected from two patients with chronic aspergillosis were included. Preliminary identification of all isolates was obtained using MALDI–ToF mass spectrometry and confirmed by AsperGenius^®^ real-time PCR assay. Antifungal susceptibility testing was determined using EUCAST E.Def 9.3 microbroth dilution. Genomic DNA libraries were constructed with the Illumina TruSeq Nano kit. Prepared whole-genome libraries were sequenced on an Illumina HiSeq 2500. Whole genome data were converted into presence/absence of a SNP with respect to the Af293 reference genome. Colonies of ARAF that co-existed in one respiratory sample demonstrated marked phenotypic diversity. Two *cyp51A* polymorphisms were found among azole-resistant isolates: TR_34_/L98H/T289A/I364V/G448S was consistently present in four variants with a pan-azole resistant phenotype and TR_34_/L98H was detected in two variants (itraconazole MIC > 16 mg/L). WGS typing showed that despite marked phenotypic variation, each sample contained a population of highly genetically related azole-resistant *A. fumigatus* variants. Our SNP analysis suggest that mechanisms additional to genetic-based variation are responsible for phenotypic diversity. Our data demonstrate that the phenotypic variants of ARAF that co-exist in clinical specimens are highly clonal and strongly suggest their origination from a single common ancestor.

## Introduction

*Aspergillus* species are saprotrophic filamentous fungi capable of causing aspergillosis. This is a broad spectrum of disease, ranging from colonization, allergic reactions (e.g., allergic bronchopulmonary aspergillosis), severe asthma with fungal sensitization, fungal ball (aspergilloma), chronic pulmonary aspergillosis (CPA), to invasive and disseminated disease in severely immunocompromised hosts [1, 2]. The cohort of patients at risk of infections by *Aspergillus fumigatus* is expanding, and newly described risk factors include prolonged stay in intensive care unit, influenza [3] or sever acute respiratory syndrome coronavirus 2 (SARS-CoV-2) infections [4, 5], and chimeric antigen receptor T-cell (CAR-T) therapy. Members of the *A. fumigatus* complex are the primary cause of human aspergillosis, however other aspergilli have become increasingly recognized as important opportunistic pathogens, such as *Aspergillus flavus* [6], *Aspergillus cladioustus* [7] and *Aspergillus nidulans* [8].

Despite recent advancements in molecular biology and implementation of nucleic-acid based tests or biomarker assays for direct detection of *Aspergillus* species in clinical specimens [9], diagnosis of various forms of aspergillosis is still largely based on isolation of aspergilli in culture. In most diagnostic laboratories, conventional identification of common species is often based on macroscopic features of typical colony combined with its classic microscopic characteristics. Additionally, matrix-assisted laser desorption/ionization-time of flight (MALDI-ToF) mass spectrometry (MS) [10] or molecular tests like *Aspergillus*-specific real-time PCR [11, 12] can be used to identify *A. fumigatus*. Sequencing of internal transcribed spacer (ITS), β-tubulin and calmodulin genes are needed to confidently separate *A. fumigatus* senso stricto from cryptic species within section *Fumigati* [13].

We observed that some respiratory samples, mainly those obtained from patients with chronic forms of aspergillosis including CPA, cystic fibrosis (CF), and aspergilloma, contain more than one phenotype (morphotype) of *A. fumigatus* concomitantly present in one sample*.* Phenotypic variation has been observed in *A. fumigatus* isolated from dogs [14], as well as among clinical isolates of *A. fumigatus* resistant to triazole antifungal agents. When compared to ‘typical’ *A. fumigatus,* unusual or abnormal phenotypes show marked variation in their growth rate, topology, reduced surface pigmentation, increased aerial hyphae, and lower sporulation (poorly- to non-sporing). Whether phenotypic variants of azole-resistant *A. fumigatus* (ARAF) that co-exist in the human airway are genetically related, or in contrast, represent a diverse and genetically distinct population that simultaneously cohabitate one biological niche is largely unexplored.

We hypothesized that phenotypic variants of azole-resistant *A. fumigatus* isolated from one clinical sample, were genetically distinct. We applied whole-genome sequencing (WGS) to examine two sets of phenotypic variants of ARAF collected from respiratory samples of two patients with chronic aspergillosis.

## Methods

### Fungal Isolates

A total of six clinical isolates of *A. fumigatus* from two patients were included in this study. First set included four isolates (CXH-01 to CXH-04) that co-existed in a single sputum sample in 2016 collected from a 73-year-old male with the clinical diagnosis of necrotizing aspergillosis. The second set, including isolates CXH-05 and CXH-06, were cultured from a single sputum specimen obtained from a 68-year-old female in 2016 with clinical diagnosis of asthma and bronchiectasis. Respiratory samples were obtained and processed as part of standard care for the patients and routine laboratory diagnosis.

### Fungal Culture and Species Identification

All samples were processed as per standard local procedures. For isolation of fungal pathogens from respiratory samples, a high-volume culture protocol previously described by Vergidis et al. [15] was followed with some modification. Briefly, equal volume of a mucolytic agent (Pro-Lab Diagnostics, Merseyside, UK) was added to each sputum and mixed thoroughly for 30 s. Samples were then incubated at room temperature for 15–20 min and mixed well prior to concentration at 3000 rpm for 10 min. The deposit was resuspended in approximately 2 mL of supernatant and 200 µL was inoculated onto centre of three Sabouraud dextrose agar plates supplemented with chloramphenicol (SABC) (Oxoid, Basingstoke, UK). Using a plate spreader, the inoculum was spread over the entire agar surface and allowed to dry for 10 min. Plates were then sealed using a gas permeable tape and incubated at 28, 35 and 45 °C ± 2 °C for up to 2 weeks and examined twice weekly for any evidence of fungal growth. All culture procedures were carried out inside a class II biological safety cabinet. Filamentous fungi were initially identified based on their macroscopic colonial features and microscopic characteristics.

Each phenotype with distinct colonial morphology were sub-cultured on SABC agar plates and incubated at 28 °C ± 2 °C for further investigation. Preliminary identification of all isolates was confirmed using MALDI–ToF MS, performed with a Microflex LT system (Bruker Daltonics, Bremen, Germany) using Biotyper 3.0 software with the additional fungi library (Bruker Daltonics, Bremen, Germany) according to the manufacturer’s recommendations. Scores of ≥ 2 were considered acceptable for species level identification. Molecular identification was carried out as previously described using AsperGenius^®^ real-time PCR assay (PathoNostics, Maastricht, Netherlands) [16].

### Antifungal Susceptibility Testing

Antifungal susceptibility testing was carried out as part of the routine diagnostic investigations and determined according to the standard EUCAST E.Def 9.3. broth microdilution method [17]. Susceptibility classification was performed according to the current EUCAST breakpoints v. 10.0 [18].

### DNA Preparation and Whole-Genome Sequencing

High molecular weight genomic DNA was extracted from all 6 isolates using the MasterPure Yeast DNA Purification Kit (Epicentre Biotechnologies) with bead beating with 1.0 mm zirconia/silica beads (BioSpec Products) in a FastPrep-24 system (MP Biomedicals) at 4.5 m/s for 45 s. Extracted DNA was quantified with a Qubit 2.0 fluorometer and dsDNA BR Assay Kit (Thermo Fisher Scientific) and quality-controled with a TapeStation 2200 and gDNA ScreenTape assays (both Agilent Technologies). Genomic DNA libraries were constructed with the Illumina TruSeq Nano kit (Illumina, San Diego, CA) at NERC Biomolecular Analysis Facility (NBAF), University of Edinburgh, Scotland, UK (http://genomics.ed.ac.uk/). Prepared whole-genome libraries were sequenced on an Illumina HiSeq 2500 sequencer at NBAF, generating 150-bp paired end reads in high output mode. Raw reads were deposited to the European Nucleotide Archive (ENA) under project accession number PRJEB27135.

### Bioinformatic Analysis

Whole-genome sequence data were analysed as previously described [19]. Briefly, all raw Illumina paired-end reads were quality checked using FastQC (v0.11.5; Babraham Institute) and aligned to the reference genome Af293 using Burrows-Wheeler Aligner (BWA v0.7.8) mem and converted to sorted BAM format using SAMtools v1.3.1. Variant calling was performed using GATK HaplotypeCaller v4.0 excluding repetitive regions identified using RepeatMaster v4.0.6. Low confidence variants were filtered out providing they met at least one of the parameters “QD < 2.0 || FS > 60.0 || MQ < 40.0 || MQRankSum < -12.5 || ReadPosRankSum < − 20.0 || SOR > 10.0”. All variant calls with a minimum genotype quality of less than 50 and not present in 90% of reads were also removed using a custom python script. Single nucleotide polymorphisms (SNPs) were mapped to genes using VCF-annotator (Broad Institute, Cambridge, MA).

Whole genome SNP data were converted into presence/absence of a SNP with respect to the reference. SNPs identified as low confidence in the variant filtration step were treated as missing data. These data were converted into relaxed interleaved Phylip format. A maximum likelihood phylogeny was constructed using rapid bootstrap analysis over 1000 replicates, and using the GTRGAMMA model of rate heterogeneity in RAxML v8.2.9; the resulting phylogeny was visualised using ggtree v3.14 [20]. All scripts used can be found at https://github.com/mycologenomics.

## Results

### Phenotypic Variation

**Fig. 1:**
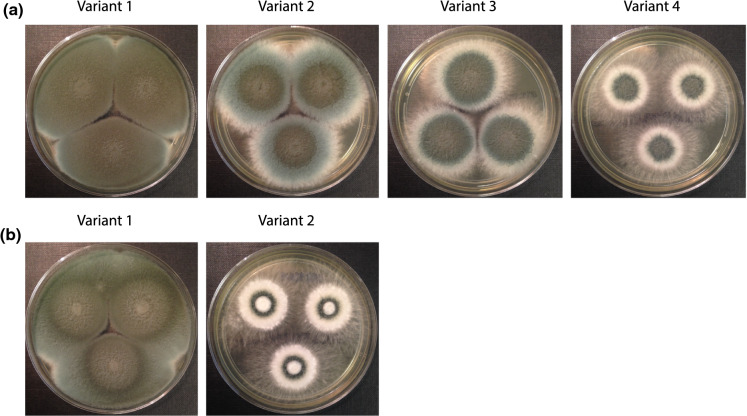
Phenotypic diversity among azole-resistant clinical *A. fumigatus* isolates. Observed colonial variants of two sets of clinical *A. fumigatus* isolates with azole-resistant profiles cultured from two sputum samples. Sabouraud dextrose agar plates were spot inoculated with conidial suspensions from each variant and incubated at 37 °C for 5 days. **a** four colonial variants CXH-01 to CXH-04 (variant 1 to variant 4; left to right) were concomitantly present in a single sputum sample from a case of necrotizing aspergillosis; **b** two colonial variants CXH-05 and CXH-06 were simultaneously cultured from a single sputum specimen obtained from a patient with asthma and *Aspergillus*-related bronchiectasis. In both panels, variants displayed a distinct phenotypic diversity potentially due to variation in their pigmentation, generation of aerial hyphae and sporulation level

As shown in Fig. 1a, four distinct phenotypes of *A. fumigatus* (CXH-01 to CXH-04) were simultaneously cultured from a single sputum sample obtained from case 1. Except variant 1 (morphologically consistent with typical *A. fumigatus*), variants 2 to 4 demonstrated marked phenotypic diversity on SABC agar after 5 days incubation at 37 °C in dark. Colonies of CXH-2 (variant 2) consisted of three clear sections: a dense central zone (dark-green), a wider zone with light green/blue pigmentation, and a peripheral area with no obvious surface pigmentation. Colonies of CXH-03 (variant 3) had an extended central zone with dark-green surface pigmentation and a wider peripheral growth without pigmentation. In contrast, colonies of CXH-04 (variant 4) showed a restricted and dense core with dark green/blue pigmentation, surrounded by a white, floccose outer zone. Notably, this variant had an aggressive and expanding peripheral growth with abundant aerial hyphae and no pigmentation on surface.

Fungal culture obtained from case 2 contained two different colonial variants, CXH-05 and CXH-06 (Fig. 1b). Colonies of CXH-05 displayed a radiant of at least three shades of green pigmentation on the surface. However, CXH-06 was phenotypically distinct from typical *A. fumigatus*. The centre of this colony was consisted of a dense non-pigmented core with restricted growth, this area was surrounded by a narrow dark-green ring, and a further floccose, non-pigmented growth. On periphery, CXH-06 (like CXH-04) showed abundant aerial hyphae and aggressive rhizoid-like outwards growth.

**Fig. 2:**
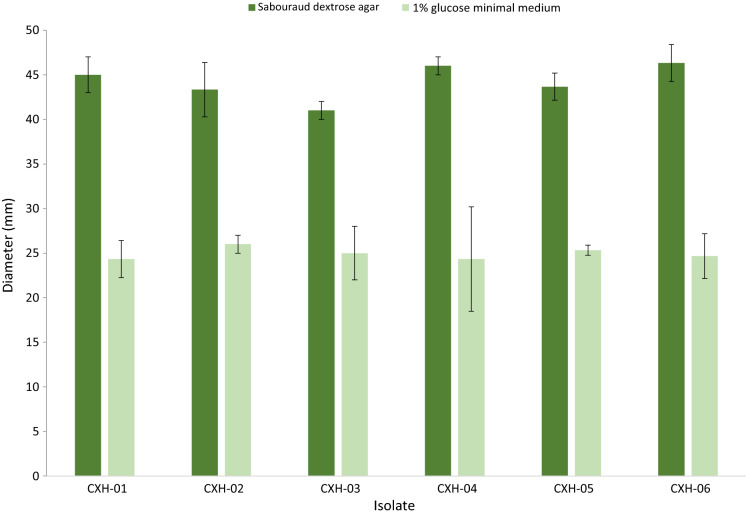
Relative radial mycelial growth rate (relative MGR). The relative MGRs are defined as the MGR of the isolates grown on Sabouraud dextrose agar and 1% glucose minimal media. Radial growth was determined by averaging the colony diameters (in mm) as measured in two randomly chosen perpendicular directions. Each isolate was tested in three technical replicates. Error bars indicate standard error of the mean (SEM). For each isolate 1,000 conidia spot-inoculated in the centre of each plate incubated at 37 °C in ambient oxygen (21% O_2_) in dark. Measurements were carried out at 72 h.

Overall, CXH-02, CXH-03 and CXH-04 (case 1) and CXH-06 (case 2), exhibited distinct phenotypes that were considered markedly different from other co-existing variants in each patient’s sample and with typical *A. fumigatus*. There was no significant difference in radial growth among these six isolates (Fig. 2). All six variants were identified as *A. fumigatus* using MALDI-ToF MS and AsperGenius® real-time PCR. Microscopically, all 6 isolates had morphological features consistent with *A. fumigatus* complex.

### Antifungal Susceptibility Profile

Susceptibility testing of isolates using EUCAST standard microbroth dilution method confirmed the phenotypic resistance to triazole antifungals in all 6 isolates (Table 1). All four variants from case 1 displayed identical antifungal susceptibility profile consistent with a pan-azole resistant phenotype. Similarly, two variants from case 2 showed identical susceptibility profiles with MIC > 16 mg/L to itraconazole. All 6 isolates remained susceptible to amphotericin B and echinocandins.

**Table 1:** Antifungal susceptibility testing results of six clinical isolates of *A. fumigatus*

Case	Isolate No	MIC (mg/L)				MEC (mg/L)		
		AMB	ITC	VRC	PCZ	MCF	ANF	CAS
1	CXH-01	0.5	16	> 16	4	0.002	0.002	0.03
	CXH-02	0.5	16	> 16	4	0.002	0.002	0.03
	CXH-03	0.5	16	> 16	4	0.002	0.002	0.03
	CXH-04	0.5	16	> 16	4	0.002	0.002	0.03
2	CXH-05	0.5	> 16	2	0.5	< 0.002	< 0.002	0.06
	CXH-06	0.5	> 16	2	0.5	< 0.002	< 0.002	0.06

AMB: amphotericin B, ANF: anidulafungin, CAS: caspofungin, ITC: itraconazole, MEC: minimum effective concentration, MIC: minimum inhibitory concentration, PCZ: posaconazole, VRC: voriconazole

### WGS and Phylogenetic Analysis

**Fig. 3:**
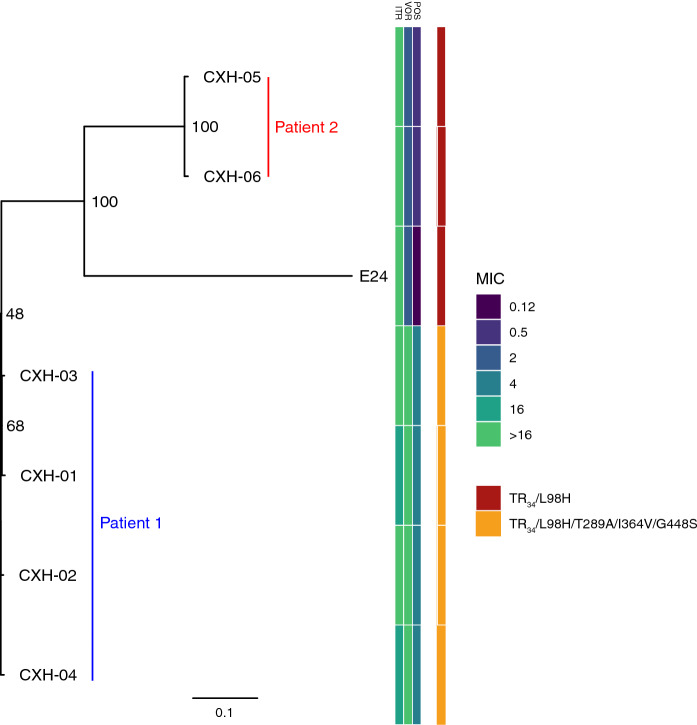
Maximum-likelihood phylogenetic tree of *A. fumigatus* isolates collected from same patient. Phylogenetic analysis reveals close genetic relatedness of isolates collected from the same patient. Isolates from patient 1 all contained the novel *cyp51A* polymorphism TR_34_/L98H/T289A/I364V/G448S, and displayed raised MICs to itraconazole, voriconazole and posaconazole. Patient 2 isolates CXH-05 and CXH-06 also displayed raised MICs to itraconazole and contained the *cyp51A* polymorphism TR_34_/L98H. Scale bar represents the mean number of nucleotide substitutions per site. Numbers on branches represent the percentage bootstrap support after 1000 iterations.

The average sequencing depth obtained for each isolate, the coverage across the genomes, number of reads aligned against Af293 reference genome, and *cyp51A* alternations are summarized in Table 2. We previously described a novel *cyp51A* genotype associated with azole-resistance, TR_34_/L98H/T289A/I364V/G448S, which was consistently detected in all four isolates CXH-01 to CXH-04. These were isolated in 2016 from a single sputum sample of a patient with necrotizing aspergillosis [19]. This novel polymorphism manifested a pan-azole resistant phenotype (Table 1), demonstrated by high MIC values for itraconazole, voriconazole (≥ 16 mg/L) and posaconazole (4 mg/L). These isolates (except for CXH-01) were phenotypically distinct from typical *A. fumigatus* displaying various colonial morphologies yet were confirmed to be *A. fumigatus* sensu stricto by mass spectrometry, real-time PCR and the subsequent WGS. All four isolates were *MAT1-2* and were only separated by 468 SNPs on average across the whole genome. A recent analysis of 218 clinical and environmental azole-sensitive and -resistant *A. fumigatus* isolated within the United Kingdom and Ireland found the average pairwise diversity to be over 11,000 SNPs [19], indicating high clonality within four azole-resistant *A. fumigatus* isolates obtained from patient 1 (Fig. 3). So far, TR_34_/L98H/T289A/I364V/G448S allele was only recovered in-patient and was not found in the environment.

**Table 2:** Details of alignments of six sequenced genomes of clinical azole-resistant *A. fumigatus* isolates from two patients. The Af293 genome was the reference genome for the number of reads aligned, the corresponding depth of coverage and the percentage of the reference genome covered by reads. Tandem repeats and SNPs with nonsynonymous substitutions in *cyp51A* gene among variants were identical among variants. Bold letters indicate alterations that are known to confer azole resistance in *A. fumigatus*

Case	Isolate No	No of reads aligned (millions)	Depth of coverage (x)	Reference genome covered (%)	*cyp51A* polymorphism
1	CXH-01	6.8	34	95.4	**TR**_**34**_, Y46F, **L98H**, M172V, N248T, D255E, **T289A**, I364V, E427K, **G448S**
	CXH-02	6.2	31	95.4	
	CXH-03	6.1	31	95.3	
	CXH-04	6.0	31	95.3	
2	CXH-05	6.1	31	95.7	**TR**_**34**_, Y46F, **L98H**, M172V, N248T, D255E, E427K
	CXH-06	6.1	31	95.7	
Control	Af293	7.0	35	99.9	–

Two azole-resistant isolates collected from patient 2, CXH-05 and CXH-06, also displayed high clonality (separated by 1461 SNPs), were both *MAT1-2*, but only contained the TR_34_/L98H *cyp51A* polymorphism. The closest unrelated isolate to those from both patients was an environmental azole-resistant *A. fumigatus* collected from South Wales, UK, which was separated on average by almost 80,000 SNPs (Fig. 3). Overall, WGS typing showed that despite marked morphologic variation among colonies of isolates that co-exist in clinical specimen, each sample contained a population of highly related azole-resistant *A. fumigatus*.

We examined the genetic relationship between isolates CXH-01 to CXH-04, and CXH-05 with CXH-06 via SNP analysis. Within the set of sequenced isolates, SNPs were distributed evenly along the genome. Unevenly distributed SNPs would suggest evolutionary pressure within a target region.

**Fig. 4:**
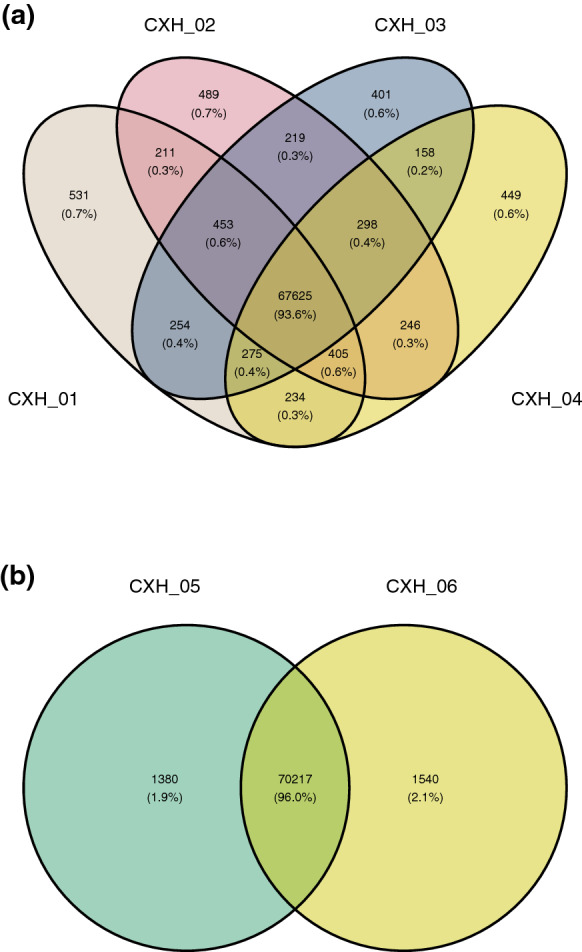
Venn diagrams of unique and common SNPs. **a** The majority of SNPs are shared between all four isolates, but more unique SNPs are found in CXH-01. **b** There are more unique SNPs found in the abnormal isolate CXH-06 when compared to CXH-05. Created using RStudio v1.4.1717 and Studio Ghibli Colour Palettes.

When compared against SNPs common to atypical isolates CXH-02, CXH-03 and CXH-04, CXH-01 had 2,363 unique SNPs (375 non-synonymous (ns) SNPs), of which 478 mapped to the coding region or intron of 47 genes. In comparison, only 298 SNPs (43 nsSNPs) were common to isolates CXH-02, 03 and 04 that are not present in CXH-01 (Fig. 4a): 58 of these SNPs mapped to the coding region or intron of 15 genes. However, different nsSNPs in CXH-01 were also found to map to 14 of these same genes, indicating that variation was occurring in these genes in both typical and atypical isolates. The single gene unique to isolates CXH-02, 3 and 4 was Afu7g01930, a putative GTP-binding protein. The 33 genes unique to CXH-01 (the normal phenotype) and absent in CXH-02, CXH-03 and CXH-4, are summarized in Table 3.

**Table 3:** Genes unique to CXH-01 containing uniquely mapped SNPs, and gene functions

Gene ID	Function
Afu1g00230	Domains with predicted ATP binding
Afu1g02450	Hypothetical protein
Afu1g04170	Domains with predicted DNA binding
Afu1g16020	Protein of unknown function
Afu2g00110	Domains with predicted transmembrane transport
Afu2g00910	NB-ARC domain protein
Afu2g03540	Orthologs have role in cell wall organisation
Afu2g05150	Putative glycophosphatidylinositol (GPI)-anchored cell wall protein
Afu2g17610	Protein of unknown function
Afu2g18100	Domains with predicted ATP binding
Afu3g03740	Putative protein kinase
Afu3g05340	Protein of unknown function
Afu3g09470	Domains with predicted coenzyme binding, oxidoreducatase activity
Afu3g10160	Domains with predicted DNA binding, RNA polymerase II transcription factor activity
Afu3g10360	Orthologs have protein transporter activity
Afu3g11960	Orthologs have RNA polymerase II core promoter proximal region sequence-specific DNA binding
Afu3g15395	Domain with predicted ATP binding
Afu4g09600	Cell wall enriched A
Afu5g03050	Orthologs have role in mRNA cis splicing
Afu5g03760	Putative class III chitinase
Afu5g12690	Orthologs have glycerone kinase activity
Afu5g12720	Putative ABC multidrug transporter
Afu5g14582	Domains with predicted catalytic activity
Afu5g14970	Protein of unknown function
Afu6g14720	Protein of unknown function
Afu7g00250	Tubulin beta-2 subunit
Afu8g04100	Putative N-acetylglucosamine-6-phosphate deacetylase
Afu8g05960	Protein of unknown function
Afu8g05980	Putative protein kinase
Afu8g06000	Domains with predicted catalytic activity
Afu8g06160	Protein of unknown function
Afu8g06210	Domains with predicted oxidoreductase activity
Afu8g06340	Protein of unknown function

When comparing CXH-06, the atypical variant, to CXH-05, we discovered 1,540 unique SNPs (Fig. 4b), of which 269 were located within the CDS or intron of genes (and 171 were nsSNPs in 26 unique genes). Five nsSNPs were located in Afu7g08250, which encodes a gene that is upregulated when conidia are exposed to neutrophils. 10 SNPs were designed nonsense or readthrough mutations, mapping to 7 unique genes which were enriched for the (1, 4)-ß-D-xylan degradation metabolic pathway (*p* < 1.48^e−2^), and xylan metabolic and catabolic GO terms (*p* < 1.63^e−2^ for both).

A recent study has identified 248 nsSNPs predicted to be involved in exposure to in-host stressors [25]. Whilst the study by Ballard et al*.* included 13 sequential isolates from one patient over 2 years, and the isolates in our study represent the diversity within a patient at one point in time, there were parallels in the findings. Whilst none of the nsSNPs identified by Ballard et al*.* were found in the isolates in this study, two genes containing nsSNPs from the Ballard et al*.* study were found to contain different nsSNPs in isolates CXH-01 to CXH-06 (Table 4). There was an incremental increase in the numbers of nsSNPs for isolates CXH-01 to CXH-04, and also CXH-5 and CXH-06, in AFUA_4G14310 (an uncharacerised protein). For gene AFUA_6G14720 (a telomere-associated RecQ helicase), increase in nsSNPs were only seen in CXH-04 (*n* = 32) compared to five nsSNPs in CXH-01 (CXH-02 and CXH-03 contains no nsSNPs in this gene). However, an increase in nsSNPs was observed for CXH-06 compared to CXH-05.

**Table 4:** Genes identified in Ballard et al*. *[25] containing nsSNPs (numbers indicated) that may be responsible for phenotypic diversity

Gene	Description	CXH-01	CXH-02	CXH-03	CXH-04	CXH-05	CXH-06
AFUA_4G14310	Uncharacterized protein	50	50	51	57	25	49
AFUA_6G14720	Telomere-associated RecQ helicase	5	0	0	32	42	52

## Discussion

To investigate the relatedness of various morphological phenotypes of azole-resistant *A. fumigatus* strains that co-existed in respiratory specimens, we sequenced the genomes of six isolates from two separate patients with chronic manifestation of pulmonary aspergillosis. Firstly, we identified a previously undescribed *cyp51A* polymorphism TR_34_/L98H/T289A/I364V/G448S in four phenotypic variants of a clinical *A. fumigatus*, which appears to be the result of recombination between genotypes containing the TR_34_/L98H, TR_46_/Y121F/T289A, and G448S polymorphisms. This polymorphism confers a pan-azole resistant phenotype, and is similar to the TR_46_^3^/Y121F/M172I/T289A/G448S polymorphism recently discovered in clinical isolates in the Netherlands [22]. Although this genotype has not yet been discovered in the environment, the occurrence of these alleles separately in environmental isolates suggests that it could have formed as a consequence of meiosis and may be recovered given further surveillance. The counterargument, that this polymorphism evolved de novo in patient as a consequence of recombination in vivo is not impossible, but is considered rare. Such de novo evolution has been observed at other regions of the *A. fumigatus* genome, notably the *hapE* gene [23]. This polymorphism was found in four clinical isolates that displayed phenotypic variation, however, it is likely not responsible for these differences observed.

Dynamics of phenotypic biodiversity in *A. fumigatus* isolates cohabitate the human airway are largely unexplored. A recent study by Valdes et al*.* [14] investigated phenotypic variation of *A. fumigatus* isolated from dogs, finding more variation than compared to clinical isolates from humans. The authors hypothesized that phenotypic variation occurred due to dog sinus inducing evolution, but they did not observe similar variation in human samples. Therefore, the identification of such variation in human samples indicates other factor(s) induce evolution.

*A. fumigatus* can exhibit extremely variable macro-morphological characteristics including sporulation, pigmentation, radial growth rate, colonial topography, and texture in primary cultures of clinical specimens. White, poorly- to non-sporing phenotypes are frequent feature of *A. fumigatus* isolates cultured from deep, usually sterile body sites [24]. Additionally, these variants are commonly seen in respiratory samples from patients with chronic cavitary pulmonary aspergillosis and CF (A. Abdolrasouli, unpublished data). The sharp morphological differences observed among colonies of *A. fumigatus* co-existed in our both patient’s samples, triggered us to believe that heterogeneous and genetically diverged populations of *A. fumigatus* were simultaneously present in each sample. Separation and characterization of each phenotype showed that, despite marked phenotypic variability among isolates, they had identical antifungal susceptibility profiles. Furthermore, WGS confirmed that four and two phenotypes obtained from first and second patients respectively, were highly clonal.

A recent study by Ballard et al*.* [25] has explored within-host evolution of *A. fumigatus*, finding certain nsSNPs have the potential to play a role in adaptation to the human host during antifungal drug therapy. Whilst we did not find the same nsSNPs identified in the Ballard et al*.* study, we did identify different nsSNPs in two of the same genes. Coupled with the additional SNP analysis completed here, it is clear that in-host adaptation involves a wide ranging selection of proteins.

Our preliminary SNP analysis based on sequencing of six isolates may also suggest that mechanisms additional to genetic-based variation are responsible for phenotypic variation. SNPs could not reliably inform about phenotypic differences; therefore, additional methods, such as RNAseq and metabolomics, may be required to elucidate these phenotypes, which are possibly due to expression of epigenetic changes. Another possible explanation lies in the number of reads that did not map to the reference genome; it is possible that phenotypic differences are due to gene loss, or that accessory genes unique to the atypical isolates are responsible.

It is possible that these phenotypically dissimilar isolates can confer different clinical outcomes. Recent research has shown that genetic diversity seen within patient can enable populations of *A. fumigatus* to adapt and also persist [26]; therefore, it is important to be characterising and sequencing multiple isolates to better understand the population of *A. fumigatus* infecting a single patient at a given time, where isolates may be genotypically similar yet phenotypically distinct. Regardless of triazole-resistance among our clinical isolates, whether their morphological diversity represents various levels of in-host adaptation requires further investigation.

Within-host diversity and evolution is seen in a number of eukaryotic pathogens, including fungi, such as *Candida* [27] and *Cryptococcus* [28] species. These genetic changes are instrumental to the maintenance of a population within a host environment [29], resulting in diverging phenotypes within a single host [30, 31]. *A. fumigatus* is no exception, undergoing niche adaptation and consequently host adaptation via the generation of genetic diversity through means such as recombination [32]. However, such phenotypic variation has rarely been seen before, possible due to a lack of adequate sampling. Currently, it is impossible to say how often this phenomenon occurs without further sampling of isolates, which is crucial for the understanding of genetic and phenotypic heterogeneities of *A. fumigatus* populations within-host. This will, importantly, have impact for comprehending virulence and the application of drug therapies.

One limitation of this study is that we did not compare the diversity of fungal isolates in clinical samples that contained mixed populations of azole-sensitive and -resistant *A. fumigatus*. Likewise, we did not examine the genetic relatedness of co-existing isolates that are morphologically indistinguishable (typical or atypical), regardless of their susceptibility or resistance to triazole antifungal drugs. In one study [33], co-existence of different genotypes of *A. fumigatus* in individual patients colonized or infected by this pathogen was shown using typing methods like random amplified polymorphic DNA (RAPD) and microsatellite analysis.

Overall, our data demonstrate that the phenotypic variants of azole-resistant *A. fumigatus* that co-exist in clinical specimens are highly clonal and strongly suggest their origination from a single common ancestor. Moreover, despite marked morphological variation, isolates showed identical susceptibility profiles. This study demonstrates the usefulness of WGS for investigation of phenotypic diversity among clinical isolates of *A. fumigatus*.

## Data Availability

Data is available under project accession number PRJEB8623 in the European Nucleotide Archive (ENA).
